# Supplementary material for: Asynchronous magnetic resonance elastography: Shear wave speed reconstruction using noise correlation of incoherent waves
Source: Magn Reson Med. 2022 Oct 27;89(3):990–1001. doi: 10.1002/mrm.29502 (PMC9792433; doi:10.1002/mrm.29502)
Supplement: Supplementary file 1 — DATA S1 MATLAB code used to generate the numerical simulations of Figure 1 [file MRM-89-990-s002.zip › k-Wave/helpfiles/waterSoundSpeed.html]

waterSoundSpeed :: Functions (k-Wave)


# waterSoundSpeed

Calculate the sound speed in distilled water with temperature.

## Syntax

```
c = waterSoundSpeed(T)
```

## Description

`waterSoundSpeed` calculates the sound speed in distilled water at a given temperature using the 5th order polynomial given by Marczak (1997, Water as a standard in the measurements of speed of sound in liquids, J. Acoust. Soc. Am., 102, 2776-2779).

## Inputs

|  |  |
| --- | --- |
| `T` | water temperature in the range 0 to 95 [degC] |

## Outputs

|  |  |
| --- | --- |
| `c` | sound speed [m/s] |

## See Also

`waterAbsorption`, `waterDensity`, `waterNonlinearity`
